# JISTIC: Identification of Significant Targets in Cancer

**DOI:** 10.1186/1471-2105-11-189

**Published:** 2010-04-14

**Authors:** Felix Sanchez-Garcia, Uri David Akavia, Eyal Mozes, Dana Pe'er

**Affiliations:** 1 Department of Computer Science, Columbia University, New York, NY, USA; 2 Department of Biological Sciences, Columbia University, New York, NY, USA; 3 Center for Computational Biology and Bioinformatics, Columbia University, New York, NY, USA

## Abstract

**Background:**

Cancer is caused through a multistep process, in which a succession of genetic changes, each conferring a competitive advantage for growth and proliferation, leads to the progressive conversion of normal human cells into malignant cancer cells. Interrogation of cancer genomes holds the promise of understanding this process, thus revolutionizing cancer research and treatment. As datasets measuring copy number aberrations in tumors accumulate, a major challenge has become to distinguish between those mutations that drive the cancer versus those passenger mutations that have no effect.

**Results:**

We present JISTIC, a tool for analyzing datasets of genome-wide copy number variation to identify driver aberrations in cancer. JISTIC is an improvement over the widely used GISTIC algorithm. We compared the performance of JISTIC versus GISTIC on a dataset of glioblastoma copy number variation, JISTIC finds 173 significant regions, whereas GISTIC only finds 103 significant regions. Importantly, the additional regions detected by JISTIC are enriched for oncogenes and genes involved in cell-cycle and proliferation.

**Conclusions:**

JISTIC is an easy-to-install platform independent implementation of GISTIC that outperforms the original algorithm detecting more relevant candidate genes and regions. The software and documentation are freely available and can be found at: http://www.c2b2.columbia.edu/danapeerlab/html/software.html

## Background

A comprehensive study of the genomic alterations that occur in cancer is vital for understanding this disease. Technological advances have made it possible to detect chromosomal regions of amplifications and deletions genome-wide and at high resolution. Datasets measuring such aberrations in patient tumors are accumulating at a staggering rate for multiple types of cancer [1-4]

However, tumors harbor a great number of copy number alterations and it is difficult to distinguish between driver aberrations (functional changes vital for cancer progression) and passenger aberrations (random and with no selective advantage). Thus, the distinction between driver and passenger mutations has become one of the key challenges in cancer genomics. A very successful algorithm to address this is "Genomic Identification of Significant Targets in Cancer (GISTIC)" [1], that identifies aberrant regions more likely to drive cancer pathogenesis. GISTIC calculates the background rate of random chromosomal aberrations and identifies those regions that are aberrant more often than would be expected by chance, with greater weight given to high amplitude events that are less likely to represent random aberrations. There are other algorithms that tackle this task such as GLAD [5], RAE [6] and STAC [6]. However, GISTIC is unique in its ability to combine magnitude and frequency of the alteration into a statistical score. This algorithm has been successfully applied to various datasets [2,4,7,8] and the approach is becoming widespread.

GISTIC identifies those regions of the genome that are aberrant more often than would be expected by chance. While successful in most scenarios, GISTIC has trouble identifying the relevant sub-region when a very large region is amplified or deleted. Such large chromosomal aberrations frequently occur in cancer and this leaves the user with two less than optimal options - consider only a single peak within the region, or consider an entire chromosomal arm. However, we have observed that in many cases there are other small sub-regions for which the aberration is significantly stronger than in the rest of the large region. Moreover, these regions often contain known oncogenes.

To address this issue, we developed JISTIC, a tool that implements all of GISTIC's capabilities, with an additional new variant of the algorithm capable of detecting multiple significant sub-regions within large aberrant regions.

## Implementation

JISTIC is based on the GISTIC algorithm [1]. JISTIC implements the previously published variants of GISTIC (standard, focal and arm-peel-off) and can also deal with LOH in the same way that the original algorithm does. More detailed information on GISTIC can be found in the Supporting Information of [1].

In brief, GISTIC calculates a statistic (G-score) which represents the strength of the aberration for each marker. The G-score for a marker m is the summation of the copy number across all samples. For this summation, the samples in which the copy number is less significant than an empirical aberration threshold (Θ^AMP ^for amplification, Θ^DEL ^for deletion) is set to zero. Therefore, the G-score in the case of amplifications is:

Where I(x) is the indicator function and CN(m, i) the copy number for marker m and sample i. The score is defined similarly for deletions, taking into account the change in sign. While standard GISTIC uses a fixed aberration threshold for each type of aberration, focal GISTIC uses sample-specific high-level thresholds. This threshold is set for each sample by adding the standard threshold to the maximum (minimum for deletions) of medians observed for each chromosome arm.

GISTIC assesses the significance of the G-score for each marker by comparing this score to results expected by chance using genome-wide permutation testing. This significance is then corrected using False Discovery Rate (FDR) using Benjamini and Hochberg method [9], and a q-value is obtained. All regions with a q-value below a threshold (0.25 in previously published articles) are deemed significant. For large aberrations, the sub-region with a minimal q-value is identified as a peak driver region.

To identify independent peaks within a region and discard spurious peaks, GISTIC uses a peel-off algorithm. The algorithm iteratively finds the most significant peak and then, for each sample, if the peak is included in the aberration, it sets to zero all markers consecutive with the peak, thus removing all aberrations overlapping the peak. Once a peak has been detected, the peak region is extended by leaving each sample out in turn, and recalculating the peak boundaries.

Typical to cancer genomes is the presence of broad copy number aberrations, defined as aberrant regions at least as large as one half of a chromosomal arm [1]. Peel-off on broad regions using standard GISTIC usually results in identifying only a single peak. GISTIC also has a focal variant of the algorithm which can potentially capture more aberrations, but misses many peaks in practice.

**Figure 1:**
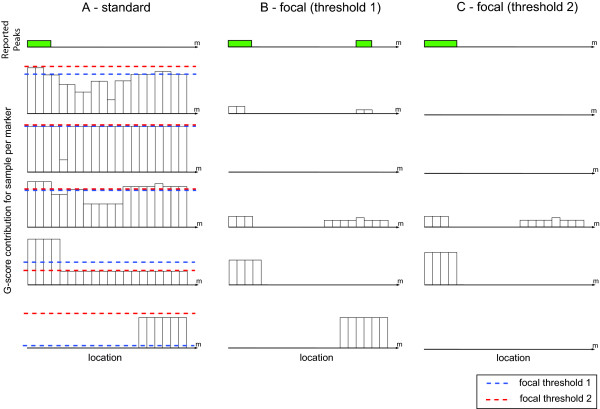
**GISTIC applied to example**. Represented is a toy chromosome for 5 samples. The X axis corresponds to consecutive markers across the chromosome and each bar represents the G-score contribution of that marker. Two possible cut-offs determined by focal GISTIC are represented by dotted blue and red lines, these cut-offs are determined based on broad regions in other chromosomes (which are not shown in the Figure for the sake of clarity). Three results from GISTIC are contemplated: standard (A), focal based on the blue threshold (B) and focal based on the red threshold (C). Reported peaks are displayed as green bars at the top, Focal GISTIC's ability to capture additional peaks is dependent on the threshold determined by aberrations in other chromosomes.

The crux of the matter is to distinguish between genuine multiple peaks and a single peak within noisy fluctuations of microarray intensities. Focal peel-off is designed to deal with this issue, but since the focal threshold is defined according to the highest broad aberration genome-wide, this variant not only requires the aberration to have a strong focal behavior, but also depends on the strength of other broad aberrations across the genome. In Figure 1 we demonstrate how different thresholds (determined by broad aberrations in other chromosomes not shown in the figure) can lead to either detection or failure to detect a second peak. In this example, standard GISTIC only captures a single peak and misses a second peak that seems equally significant, with just a slight difference in G-score. In this type of situations standard GISTIC is doomed to fail. The detection of the second peak with focal GISTIC highly depends on the focal threshold. This threshold is set for each sample by the highest broad aberration genome-wide and there are cases, such as C in Figure 1, in which the focal events that define the aberration are masked by broad aberrations in other chromosome (not shown in Figure). In the next section we will see that this problem is prevalent in real datasets.

For JISTIC, we developed a new variant called limited peel-off, designed to tackle the problem posed in Figure 1. While GISTIC sets to zero any aberration containing the peak, our algorithm "peels off" only part of the aberration. Limited peel-off uses the global G-score to determine the extent of "peel-off. Given a single aberration, we expect G(m) would decrease as we get further from it until it reaches the noise level. The idea behind limited peel-off is to decompose the G-score for a marker in two parts, one that represents what remains from the peak that we are peeling off (G_r_(m)) and another that depicts contribution independent of the peak (G_n_(m)). We use a threshold value (G_thres_) to estimate whether G_n_(m) contains only noise or the aberration is likely due to an independent peak.

Denote **G(m, i) **as the G-score contribution of sample i in marker m, which can be defined as:

For each sample i, the algorithm iteratively calculates on both sides of a newly discovered peak the magnitude **G_r_(m, i)**. For the right side of the peak:

the left side of the peak is the symmetric equation:

G_r_(m, i) represents the amount of aberration that remains from the primary peak in marker m. JISTIC considers any reduction in the aberration that is consistent for at least a minimal number of markers, s. This parameter was introduced after observing that transient fluctuations in copy number result in a greater number of false positives. For s = 0 a total of 147 peaks are detected with limited peel-off. However, many of those peaks are located next to each other in the genome and the biological assessment suggested that those were spurious peaks. The number of reported peaks decreases when the parameter s increases, eliminating most of those spurious peaks.

In order to determine whether a new peak exists, we need the complementary component of G_r_(m, i), G_n_(m, i), measuring the component of the aberration that is independent from the primary peak, for each sample:

The condition that JISTIC checks in order to abort the peel-off at a marker m is

The results critically depend on the threshold G_thres_. If G_thres _is too high, the algorithm will obtain exactly the same result than the standard variant of GISTIC. If the value is too low, the algorithm will obtain many spurious peaks. For consistency, we use the G-score value that corresponds to the threshold q-value when running the focal variant, thus using the same threshold both for a peak on baseline copy number and a sub-peak within a broader region.

**Figure 2:**
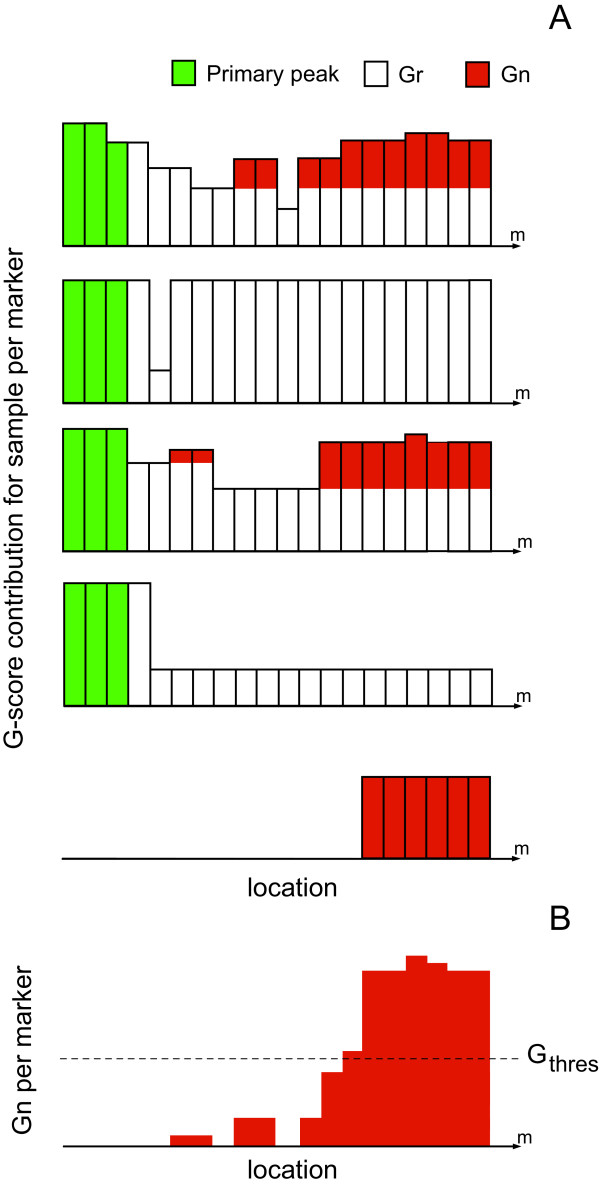
**Limited peel-off applied to example**. Limited peel-off for the same example shown in Figure 1. The x axis corresponds to consecutive markers across the chromosome while each bar represents the G-score contribution of that marker. Reported peaks are displayed as green bars at the top. Limited peel-off successfully detects two peaks in the example, independently of aberrations in other chromosomes. The figure also illustrates how thresholds in limited peel-off are applied. In this example s = 1. **(A) **The G-score contribution for each sample is decomposed into the G-score for the primary peak (green), G_r _(white) and G_n _(red). **(B) **Each bar illustrates the total G_n _of the marker. The threshold G_thres _represents the cut-off that determines whether the peel-off should be aborted. In this example the peel-off is aborted in marker 14, allowing the detection of an additional peak.

Figure 2 shows how limited peel-off successfully detects the second peak in the example from Figure 1. Contrary to the focal variant, this success is independent of broad regions in other chromosomes. It is important to note that limited peel-off fully replaces the standard variant, as any peak captured by the latter, will also be captured by the former. On the other hand, focal and limited peel-off can be considered complementary. While limited peel-off looks at the average of the aberration across samples, focal concentrates on the most acute aberrations for each sample.

## Results

We tested JISTIC on 178 glioblastoma samples that had been previously used to test the latest version of GISTIC [10]. This dataset was generated by Harvard Medical School using Agilent 244 K Arrays.

The parameter s was tuned using the histogram of segments sizes to obtain candidate values and the optimal value was chosen based on the distribution of minimum distance between peaks. In the test dataset, the histogram of segment size showed that the number of segments decreases with the segment size, as it was expected. We observed a stronger decrease in the number of segments for 3 different segment sizes (3, 8 and 12); those sizes were considered as candidate values for s. The distribution of minimum distance between peaks was used to estimate the number of spurious peaks for each of those values. This distribution shows a considerable change when s was increased to 8, as the number of adjacent peaks decreased. The distribution did not show any significant change when s was increased to 12 and we adopted a conservative approach by setting s = 10, which corresponds to 126.87 kb on average. Limited peel-off detected 124 peaks using s = 10.

Table 1 shows the peaks and genes obtained with JISTIC in the three variants: standard, focal and limited peel-off. Note that standard and focal are equivalent to implementations of the GISTIC algorithm [10]. When looking at peaks by chromosome (Table 2), we observe that most of the novel peaks appear in chromosomes with a broad region for which standard detects only a single peak and focal detects one or no peaks.

**Table 1:** Summary of peaks and genes for the three variants.

	Amplification		Deletion	
		
	Peaks	Genes	Peaks	Genes
**Standard**	14	35	12	59

**Focal**	74	202	21	53

**Standard+ Focal**	75	204	28	99

**Limited peel-off**	64	272	60	205

**Novel by limited peel-off**	29	186	41	136

For each variant and type of aberration, the number of peak regions and genes contained within them are illustrated. The table also summarizes results for all GISTIC variants (non-overlapping peaks obtained by standard and focal GISTIC) and novel peak regions reported by limited peel-off. Limited peel-off detects 70 novel peaks and 322 genes.

**Table 2:** Peaks by chromosome.

		1	2	3	4	5	6	7	8	9	10	11	12	13	14	15	16	17	18	19	20	21	22
**AMP**	**Standard**	2	0	1	4	0	0	**3**	0	0	0	0	2	0	0	0	0	0	0	**1**	**1**	0	0
	
	**Focal**	9	6	5	6	2	3	**17**	1	0	0	3	16	1	1	1	0	1	0	**0**	**1**	0	1
	
	**Limited**	6	0	2	4	0	0	**28**	0	0	0	0	13	0	0	0	0	0	0	**7**	**4**	0	0

**DEL**	**Standard**	1	0	0	0	1	**1**	0	0	**1**	**2**	1	0	**1**	**1**	1	0	0	0	1	0	0	**1**
	
	**Focal**	4	1	0	1	2	**1**	0	1	**7**	**1**	0	0	**1**	**1**	0	0	0	0	1	0	0	**0**
	
	**Limited**	5	0	0	0	1	**6**	0	0	**16**	**12**	1	0	**7**	**4**	3	0	0	0	1	0	0	**4**

**Figure 3:**
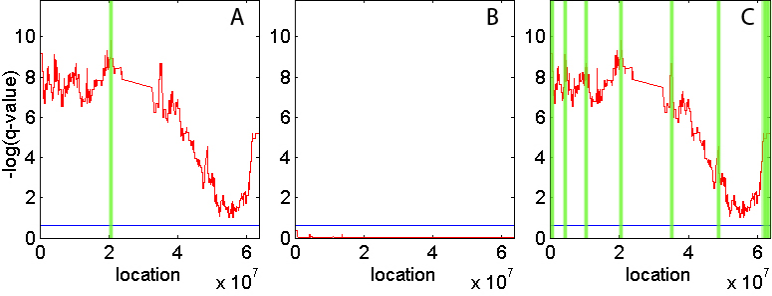
**Amplification aberrations for chromosome 19**. Represented are standard (A), focal (B) and limited peel-off (C) results for amplifications in the whole chromosome 19. The x axis corresponds to consecutive markers across the chromosome and the y axis corresponds to the q-value in logarithmic scale. The significance threshold for aberrations (q-value = 0.25) is represented by a blue line. Reported peak regions are illustrated in green. Limited peel-off detects 6 novel peaks.

Figure 3 demonstrates an example of such a broad peak in chromosome 19. Standard (A) captures only a single peak and focal (B) fails to capture any aberration. On the other hand, limited peel-off (C) successfully captures multiple relevant regions. The ultimate assessment is whether the additional peaks identified are indeed drivers. To evaluate this we assessed whether the new peaks in chromosome 19 contained solid candidates for oncogenes. Standard GISTIC identified a single peak containing the gene (ZNF626), whose role is unknown. In comparison, limited peel-off detected 7 additional regions, where each includes at least one oncogene and multiple genes related to cell replication. Examples of oncogenes include AP1 M2 [11], Cyclin E1 [12], LYPD3 [13], see Table 3 for list of potential oncogenes in each peak.

**Table 3:** Novel amplification peaks in chrom. 19 reported by limited peel-off.

Location	Candidate driver genes
**265456-781585 (19p13:3)**	PTBP1 [21], BSG [22]

**4105670-4353981 (19p13:3)**	CHAF1A [23], SH3GL1 [24]

**10539157-10628316 (19p13:2)**	AP1 M2 [11], ILF3 [25]

**34895742-35531840 (19q12)**	Cyclin E1 [12]

**48658163-48753325 (19q13:31)**	LYPD3 [13]

**61586503-63564951 (19q13:43)**	BMZF3 [26]

For each novel peak region of amplification detected by limited peel-off in chromosome 19 its location in the genome and suspected driver genes are shown. Each driver gene is accompanied by a reference that supports its oncogenic nature. All novel peaks contain at least one candidate driver gene.

**Figure 4:**
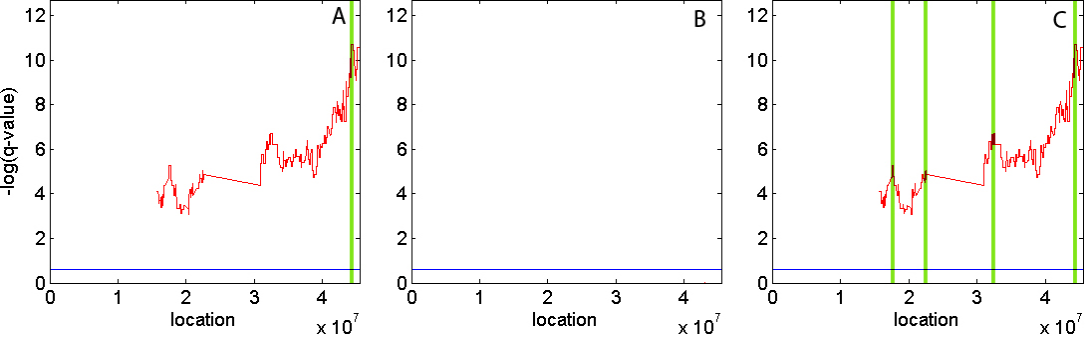
**Deletion aberrations for chromosome 22**. Represented are standard (A), focal (B) and limited peel-off (C) results for deletions in the whole chromosome 22. The x axis corresponds to consecutive markers across the chromosome and the y axis corresponds to the q-value in logarithmic scale. Note that only markers for which data is available are plotted. The significance threshold for aberrations (q-value = 0.25) is represented by a blue line. Reported peak regions are illustrated in green. Limited peel-off detects 3 novel peaks.

Figure 4 shows another example, a deletion of the entire chromosome 22. Standard finds a single peak and focal finds no peaks, whereas limited peel-off finds 3 additional peaks. The peak identified by standard includes FBLN1, a gene that has been previously selected as candidate tumor suppressor [14]. Each additional peak identified by limited peek-off has at least one tumor suppressor, such as SMARCB1 [15], HIRA [16] and LARGE [17], see Table 4 for list of potential tumor suppressors in each peak.

**Table 4:** Novel deletion peaks in chrom. 22 reported by limited peel-off.

Location	Candidate driver genes
**17482013-17787269 (22q11:21)**	HIRA [16]

**22370186-22476593 (22q11:23)**	SMARCB1 [15]

**32114340-33392384 (22q12:3)**	LARGE [17]

For each novel peak region of deletion detected by limited peel-off in chromosome 22 its location in the genome and suspected driver genes are shown. Each driver gene is accompanied by a reference that supports its behavior as tumor suppressor. All novel peaks contain at least one candidate driver gene.

For a systematic evaluation we designed an automated statistical test to study the limited peel-off's specificity and establish that the increased number of peaks detected by limited peel-off does not increase the false positive rate. Our permutation based test estimates the number of candidate cancer driver genes expected in random regions matching in size with the regions detected by JISTIC. We compiled a list of 1880 likely driver genes based on two different sources:

• Genes listed in the Sanger Cancer Gene Census [18].

• Genes annotated in GO [19] for at least one of the following processes: cell cycle, cell proliferation, cell death and neurogenesis.

We generated 10,000 random outputs with similar characteristics to the real output. A random output is generated by iterating over all detected peaks and shifting the peak's location to a random position in the genome. For each output, we count the number of regions containing at least one gene in the list of driver candidates above. This provides us with a distribution for the number of potential driver genes expected in the null model.

To compare focal and limited peel-off, we tested three sets of peaks: the peaks that are exclusive to each of the two methods and the peaks that are common to both. Note that all the peaks obtained by standard GISTIC are also obtained by limited peel-off. The results of the 10,000 iterations for each of the three sets are shown in Table 5.

**Table 5:** Results for permutation test for focal and limited peel-off peaks.

	Peaks	Driver Peaks	P-value
**Focal exclusive**	49	9	7.3E-3

**Limited peel-off exclusive**	78	25	2.3E-3

**Overlapping between both**	46	14	3.5E-6

The p-value for the permutation test estimates the probability of detecting as many peaks containing candidate driver genes if random regions of the same size across genome are selected. The table shows results for mutually exclusive peaks between focal GISTIC and limited peel-off and for overlapping peaks between both variants.

As expected, the p-value obtained for the overlapping peaks is more significant than the p-value for the exclusive peaks for each method.

For the 78 peaks obtained exclusively by limited peel-off, 25 contained likely driver genes, only 23/10,000 permutations reached the value obtained by the true output, giving a p-value of 0.0023. In comparison, for exclusive peaks for the focal variant, 9 of the 49 peaks contained likely driver genes. In 73/10,000 permutations the test obtained as many peaks with likely driver genes, giving a p-value of 0.0073.

## Conclusions

The analysis performed on limited peel-off against standard and focal GISTIC demonstrates the superiority of limited peel-off to achieve both better specificity and dramatically increase recall by obtaining a large number of novel peaks.

In conclusion, JISTIC is a significantly improved algorithm to distinguish between driver and passenger copy number aberrations in cancer genomes. Importantly, it detects a significant number of additional driver regions while maintaining a similar false positive rate. We conclude that both limited peel-off and focal GISTIC should be used together as they provide complementary and significant results.

The tool is implemented in Java, has been tested on Linux and Windows. It can be downloaded from http://www.c2b2.columbia.edu/danapeerlab/html/software.html. It does not have dependencies to external libraries and can be downloaded as a single Java JAR file. The execution time for the glioblastoma dataset on a standard desktop computer (Intel Xeon W3505 @ 2.53 GHz, 3GB of RAM) was 8 minutes for all variants.

**Figure 5:**
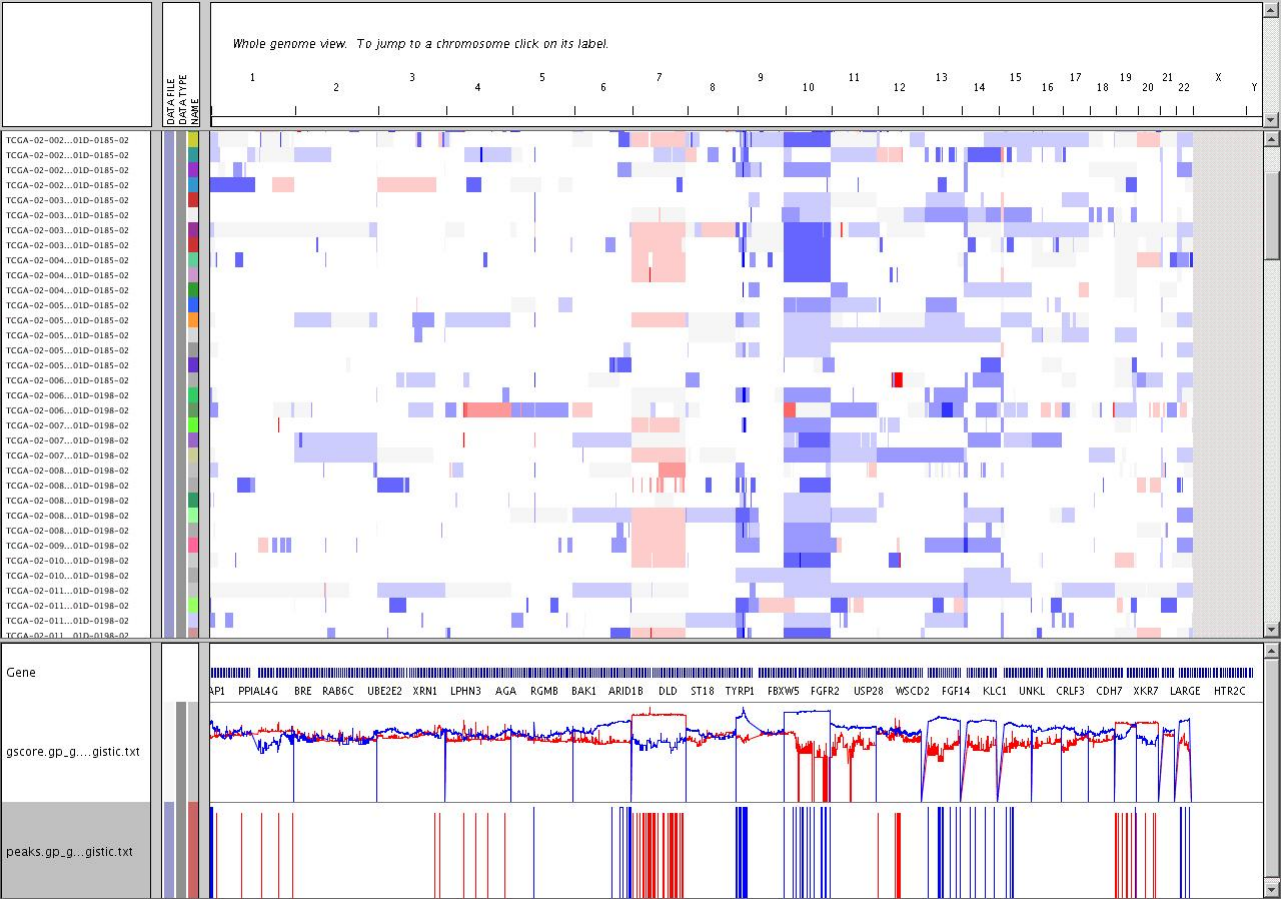
**IGV display of JISTIC results**. Segmented copy number and JISTIC results can be simultaneously displayed using the visualization tool IGV [20]. The main window displays the segmented copy number data used as JISTIC input. Two tracks at the bottom show JISTIC's output (G-score and peak regions respectively).

Matlab scripts are provided in order to visualize the output and obtain different statistics. The tool can also convert to the format used by the open-source visualization tool IGV [20], used to display cancer genomic data using a user-friendly interface, demonstrated in Figure 5.

## Availability and Requirements

• **Project name**: JISTIC

• **Project home page**: http://www.c2b2.columbia.edu/danapeerlab/html/software.html

• **Operating system(s): **Platform independent

• **Programming language**: Java

• **Other requirements**: Java 1.5 or higher

• **License**: LGPL

• **Any restrictions to use by non-academics**: None

## Conflict of interests

The authors declare that they have no competing interests.

## Authors' contributions

DP, FSG and UDA designed research. FSG and UDA developed the limited-peel off algorithm EM and FSG implemented the JAVA code. FSG and UDA analyzed and evaluated the algorithm. FSG and DP wrote the manuscript. All authors read and approved the final manuscript.
